# Screening and Characterization of Purine Nucleoside Degrading Lactic Acid Bacteria Isolated from Chinese Sauerkraut and Evaluation of the Serum Uric Acid Lowering Effect in Hyperuricemic Rats

**DOI:** 10.1371/journal.pone.0105577

**Published:** 2014-09-03

**Authors:** Ming Li, Dianbin Yang, Lu Mei, Lin Yuan, Ao Xie, Jieli Yuan

**Affiliations:** 1 Department of Microecology, School of Basic Medical Science, Dalian Medical University, Dalian, Liaoning, China; 2 Department of Gastroenterology, the Second Affiliated Hospital of Zhengzhou University, Zhengzhou, Henan, China; 3 Faculty of Agricultural, Life and Environmental Sciences, University of Alberta, Edmonton, Alberta, Canada; Friedrich-Alexander University Erlangen, Germany

## Abstract

Hyperuricemia is well known as the cause of gout. In recent years, it has also been recognized as a risk factor for arteriosclerosis, cerebrovascular and cardiovascular diseases, and nephropathy in diabetic patients. Foods high in purine compounds are more potent in exacerbating hyperuricemia. Therefore, the development of probiotics that efficiently degrade purine compounds is a promising potential therapy for the prevention of hyperuricemia. In this study, fifty-five lactic acid bacteria isolated from Chinese sauerkraut were evaluated for the ability to degrade inosine and guanosine, the two key intermediates in purine metabolism. After a preliminary screening based on HPLC, three candidate strains with the highest nucleoside degrading rates were selected for further characterization. The tested biological characteristics of candidate strains included acid tolerance, bile tolerance, anti-pathogenic bacteria activity, cell adhesion ability, resistance to antibiotics and the ability to produce hydrogen peroxide. Among the selected strains, DM9218 showed the best probiotic potential compared with other strains despite its poor bile resistance. Analysis of 16S rRNA sequences showed that DM9218 has the highest similarity (99%) to *Lactobacillus plantarum* WCFS1. The acclimated strain DM9218-A showed better resistance to 0.3% bile salt, and its survival in gastrointestinal tract of rats was proven by PCR-DGGE. Furthermore, the effects of DM9218-A in a hyperuricemia rat model were evaluated. The level of serum uric acid in hyperuricemic rat can be efficiently reduced by the intragastric administration of DM9218-A (*P*<0.05). The preventive treatment of DM9218-A caused a greater reduction in serum uric acid concentration in hyperuricemic rats than the later treatment (*P*<0.05). Our results suggest that DM9218-A may be a promising candidate as an adjunctive treatment in patients with hyperuricemia during the onset period of disease. DM9218-A also has potential as a probiotic in the prevention of hyperuricemia in the normal population.

## Introduction

Hyperuricemia is caused by abnormally high level of uric acid in the blood [1]. It is known to cause the formation of solid uric acid crystals within joints, which results in a painful condition commonly known as gout [2]. In recent years, hyperuricemia has also been recognized as a risk factor for arteriosclerosis, cerebrovascular and cardiovascular diseases [3], chronic kidney disease [4] and nephropathy in diabetic patients [5], [6]. Several studies have reported that experimentally induced hyperuricemia in rodents can cause metabolic syndrome, hypertension and renal disease [7]–[9]. Lowering uric acid was proven to improve renal function, proteinuria and tubulointerstitial damage in type 2 diabetic *db/db* mice. The mechanism is likely caused by blocking uric acid-induced intrarenal inflammation [10].

Factors contributing to hyperuricemia vary from genetics, insulin resistance, hypertension and renal insufficiency, to obesity, diet and the consumption of alcoholic beverages [11]. Foods high in the purines adenine and hypoxanthine are more potent in exacerbating hyperuricemia [12]. Therefore patients with hyperuricemia have to strictly control their diet. However, accurate information on which food products and which nutrients affect plasma uric acid concentration are limited, and thus the dietary recommendations are currently unclear [13]. In addition, the purine compounds disodium 5′-guanylate and disodium 5′-inosinate are the main components of many flavor enhancers that are widely used in modern food production; thus, a strict diet means the changing both food flavors and eating habits. Drugs used to treat hyperuricemia are effective but with many side-effects. For example, allopurinol can induce hypersensitivity syndrome (AHS), which may lead to the death of patients [14]. In comparison to restricting the diet, the use of purine compound degrading probiotics is a promising alternative for the prevention of hyperuricemia.

Lactic acid bacteria (LAB) are Gram positive, acid-tolerant, fermenting rods or cocci that produce lactic acid as the major metabolic end-product of carbohydrate fermentation [15], [16]. They are used in the manufacture of dairy products such as acidophilus milk, yogurt, cheeses and pickled vegetables. Numerous LAB strains have been continuously screened for desirable characteristics, such as stimulation of immune system [17], antitumor activity [18], stabilization of gut microbiota [19] and inhibition of pathogenic species. These beneficial properties make LAB strains valuable as probiotics, and many of them are used as starter microorganisms in yogurt fermentation [20]. However, no serum uric acid-lowering LAB and systemic evaluation of the probiotic characteristics have been reported.

In this study, we report for the first time the screening of purine nucleoside degrading LAB strains isolated from Chinese sauerkraut, and evaluate the probiotic characters of selected candidate strains. The effects of the optimal strain on a hyperuricemia rat model were also presented. We believe that our results will provide a reference for the development of hyperuricemia-preventing probiotics.

## Materials and Methods

### Isolation of LAB

To isolate LAB, different fresh Chinese sauerkrauts were purchased from local markets in Dalian, China. Each sample (10 g) was blended with 50 mL of 0.85% NaCl solution and further diluted in a 10-fold dilution series with 0.85% NaCl solution (10^−1^∼10^−8^). Each diluted solution was spread-plated onto de Man, Rogosa and Sharpe (MRS) agar (Difco, USA). The plates were incubated in anaerobic chest with AnaeroPack (Beijing B-Y Tech Co., Inc., China) at 37°C for 48 h. The bacterial colonies appeared on the plates were picked and streaked on fresh MRS agar plates. Single colonies were again picked and stored in 20% glycerol at −80°C. The isolates were screened for catalase activity and Gram staining, and only those that were catalase-negative and Gram-positive were selected for further studies. Prior to experiments, the stocks were propagated twice in MRS broth at 37°C for 24 h.

### Screening of guanosine and inosine degrading LAB strains

A HPLC solution system was used to detect both inosine and guanosine simultaneously. The procedure is as follow: 33.7 mg of inosine and 35.7 mg of guanosine was dissolved in 100 mL K_3_PO_4_ solution (100 mmol/L, pH = 7.0) to make inosine-guanosine solution. After filtration (0.22 µm), 5, 10, 15, and 20 µL inosine-guanosine solutions were injected into a HPLC device (LC-20A, Shinadzu Corporation, Japan) equipped with a variable wavelength detector and a Cosmosil-5C_18_-AR-II column (4.6×250 mm, Cosmosil, Japan). The isocratic elution was performed with a NaClO_4_-H_3_PO_4_ solution (0.1 µmol/L NaClO_4_, and 0.187 mol/L H_3_PO_4_ in dH_2_O), at flow rate of 1 mL/min. Contents of inosine and guanosine were identified at 254 nm by retention time of 14.906 and 10.889 min, respectively, and quantified by interpolation of calibration curves. The deduced standard curves were A_ino_ = 5×10^7^C_ino_+22844, R = 0.9999, and A_gua_ = 5×10^7^C_gua_−10822, R = 0.9999. A: the peak area, C: concentration (g/L).

To evaluate the inosine and guanosine assimilating ability, LAB strain was inoculated in MRS and cultured for 48 h at 37°C under anaerobic conditions. 2 mL of the culture broth was centrifuged at 4,000×g, 4°C for 10 min. The cells were then washed twice with 1 mL 0.85% NaCl, resuspended with 750 µL of inosine-guanosine solution and incubated at 37°C for 60 min, with shaking (120 rpm). After that, the solution was centrifuged at 4,000×g, 4°C for 10 min. 270 µL of the supernatant was removed. 30 µL HClO_4_ (0.1 mol/L) was added into the supernatant, mixed thoroughly to prevent further degradation. 20 µL of the mixture was injected into the HPLC device after filtration. The remaining inosine and guanosine contents were calculated by the formula deduced above. The degrading speed and rate of inosine or guanosine by different LAB strains were calculated according to the following formula: *V* = (0.9C-X)/60, α = [(0.9C-X)/0.9C]•100%. *V*: degrading speed (g/L/min), X: the remaining content of inosine or guanosine (g/L).

To evaluate the degradation of purine compounds by cell-free extracts of LAB, 2 mL of the culture broth was centrifuged at 4,000×g, 4°C for 10 min. The cells were then washed twice with 1 mL 0.85% NaCl, resuspended with 750 µL of inosine-guanosine solution and were sonicated as described by Feliu *et al* [21]. After that, the extracts were incubated at 37°C for 60 min and 120 min, with shaking (120 rpm). The contents of inosine and guanosine were analyzed by HPLC as mentioned above.

### Characterization of candidate strains as potential probiotics

#### Acid tolerance

Resistance to acidic conditions was tested by inoculating 10^8^ CFU/mL (final bacterial concentration) of LAB in MRS broth with different pH values [22]. The pH of MRS was adjusted to 2.0, 3.0 and 4.0 using 0.1 N HCl. After cultivation for 4 h at 37°C, serial dilutions were performed and the growth of the bacterial strains was recorded by viable plate counting. This assay was performed in triplicate.

#### Bile tolerance

The bile salt solutions were prepared using ox gall powder (Sigma, St. Louis, Mo, USA) at final concentrations of 0.1%, 0.2% and 0.3% [23]. 10^8^ CFU/mL (final bacterial concentration) of freshly prepared LAB were inoculated into 10 mL of the autoclaved solutions and incubated at 37°C for 4 h before viable plate counting. This assay was performed in triplicate.

#### Tolerance to pepsin and trypsin

10^8^ CFU/mL (final bacterial concentration) of freshly prepared LAB cultures were inoculated into MRS broth with different concentrations of pepsin (0.59 µg/mL, 0.72 µg/mL, and 1.48 µg/mL), and trypsin (0.336 µg/mL, 0.592 µg/mL, and 0.723 µg/mL). Growth of strains was enumerated 4 h after incubated at 37°C. This assay was performed in triplicate.

#### Test of antimicrobial activity

To evaluate the antimicrobial ability of the isolates, agar spot test was performed [22]. The pathogenic bacteria used in this study are *Escherichia coli* ATCC 8739, *Staphyloccocus aureus* ATCC 6538P, *Micrococcus luteus* MTCC 2470, *Salmonella typhi* ATCC 786, *Pseudominas aeruginosa* ATCC 25619, and *Staphylococcus epidermidis* ATCC12228. Briefly, 2.5 µL of a fresh LAB culture was spotted on MRS agar plates and incubated at 37°C for 24 h. After that the agar surface was covered with 15 mL of LB agar inoculated with the pathogenic strains at concentration of 10^6^ CFU/mL. The plates were then incubated at 37°C. Inhibition of growth was detected after 24 h by measuring the diameters of inhibition zones. The test was performed in triplicate.

#### Measurement of H_2_O_2_ production

The freshly cultured cells of LAB strains were harvested by centrifugation at 10,000 rpm for 10 min at 0°C. 2 g of the cell pellet was resuspended in 20 mL of cold, sterile phosphate buffer (adjusted to pH 6.5). The cells were then incubated at 5°C for a period of 5 days under anaerobic condition. Measurements of H_2_O_2_ were done according to the methods of Villegas and Gilliland [24].

#### Cell adhesion ability

The adhesion ability of candidate strains to the human enterocyte-like Caco-2 cells was evaluated using the method described previously [29]. Briefly, the cells were grown in Dulbecco's minimal essential medium (DMEM) (Invitrogen, Germany) with 100 U/mL penicillin and 100 mg/mL streptomycin. Before the adherence assays, Caco-2 cells were cultured in 2 mL of the medium without antibiotics for 10 days. After standardization of conditions and preparation of monolayer (1×10^5^ cells/well) of cell lines, 1 mL of the medium was replaced with 1 mL of LAB suspension (10^8^ CFU/mL in DMEM). The inoculated cultures were then incubated for 3 h at 37°C in 5% CO_2_. The infected cells were washed 3 times with sterile PBS (pH 7.8), fixed for 2 h with 10% formaldehyde, Gram-stained, and observed microscopically (1,500×magnification, with oil immersion). The adherent bacteria from 20 randomly selected microscopic fields were counted. All samples were analyzed in triplicate.

#### The sensibility of the strains to the antibiotics

Disk diffusion susceptibility tests were performed according to Clinical and Laboratory Standards Institute (CLSI) standard procedure [25]. Lab strains were inoculated in MRS and incubated at 37°C for 24 h. The culture broth was then diluted to a concentration of 6×10^8^ CFU/mL, and spreaded onto the entire surface of a dried MRS agar plate using a sterile cotton swab. Antibiotic discs (see contents of antibiotics in Tab. 3) were placed on the surface of each MRS plate. After incubation for 48 h at 37°C, the diameter (in mm) of the inhibition zone around each disk was measured to classify the antibiotic sensitivity of each isolate. All samples were analyzed in triplicate.

**Table 3:** The Susceptibility of candidate LAB strains to different antibiotics.

Category	Antibiotics	Dose (µg/disc)	Susceptibility		
			DM9218	DM9242	DM9505
Penicillins	Penicillin (P)	10 IU	**I**	**S**	**S**
	Ampicillin (AMP)	10	**I**	**S**	**I**
	Piperacillin (PRL)	100	**S**	**S**	**S**
	Amoxicillin (AMX)	10	**S**	**S**	**S**
Cephalosporins	Cefazolin (CZ)	30	**I**	**S**	**I**
	Cefalotin (KF)	30	**S**	**S**	**S**
Aminoglycosides	Gentamycin (CN)	10	**R**	**R**	**R**
Quinolones	Levofloxacin (LE)	5	**R**	**R**	**R**
	nalidixic acid (NA)	30	**R**	**R**	**R**
	Ciprofloxacin (CIP)	5	**R**	**R**	**R**
Macrolides	Azithromycin (AZM)	15	**I**	**S**	**I**
	Erythromycin (E)	15	**S**	**S**	**S**
Tetracyclines	Tetracycline (TE)	30	**I**	**S**	**I**
Ansamycins	Rifampicin (RD)	5	**S**	**S**	**S**
Glycopeptides	Vancomycin (VA)	30	**R**	**R**	**R**
Combination	Sulfamethoxazolum-Trimethoprimum (SXT)	23.75–1.25	**I**	**I**	**S**

S, susceptible, the diameter of inhibition zone ≥17 mm; I, intermediate, the diameter of inhibition zone between 12–17 mm; R, resistant, the diameter of inhibition zone ≤12 mm resistance.

### Identification of strain DM9218

The 16S rDNA sequence was amplified using the primers described by Dubernet *et al* [26]. The sequence data was submitted to GenBank of NCBI, under accession number KF753248 and compared with the similar reference species using BLAST program, which is available at http://www.ncbi.nlm.nih.gov/. Phylogenetic tree was constructed based on the 16S rRNA gene sequences using Neighbor-joining method by MEGA 6.0 program.

### DGGE analysis and 16S rDNA sequencing

The metagenomic DNA was extracted from the frozen feces of randomly selected rats (7/group) by the QIAamp DNA stool mini kit (Qiagen, Germany). PCR was conducted using universal primers F338+GC clamp and R518 targeting the hyper variable V3 region of 16S rRNA gene [27]. The resulting 16S rDNA amplicons were analyzed using the DCode system (Bio-Rad, USA) according to descriptions of Joossens *et al* [27]. Fragments of interest were excised from the gel and macerated, and the suspension was incubated for 10 min at 98°C. The supernatant was used in PCR with F338 and R518 primers. The obtained PCR products were purified using the QIAquick PCR purification kit (Qiagen, Germany) and send for sequencing (Takara, Japan).

### Animal test

The study protocol was approved by the Animal Care Committee of the Dalian Medical University, China (SCXK-2008-0002). 40 male Wistar rats, aged 30 days, were obtained from the SPF animal center of Dalian Medical University. They were divided randomly into 5 experimental groups (8/group). The control group was given food and water *ad libitum*. The hyperuricemia group, the allopurinol group, and the DM9218-After group were first treated with high purine diet for 7 days. The high purine diet (per 100 g) contains 87 g yeast extract (Sigma, USA), and 1.5 g ribonucleic acid from torula yeast (R6625, Sigma, USA). The DM9218-Before group was treated with high purine diet and DM9218-A cell solution (1.2×10^9^ CFU/mL in 0.85% NaCl, 1 mL per day) for 7 days. On the 8^th^ day, except the control group, other groups were injected intraperitoneally by potassium oxonate (0.35 mg/100 g body weight per day)-carboxymethylcellulose sodium (CMC-Na) solution (3 g/L) for 7 days. Meanwhile, the allopurinol group was administrated with 4.2 mg/100 g (body weight) of allopurinol (Shenyang No. 1 Pharmacheutical Co. LTD, China) per day, and the DM9218-After group was treated with DM9218-A cell solution (1.2×10^9^ CFU/mL in 0.85% NaCl, 1 mL per day) for 7 days. The rat body weight was recorded randomly during the 14 days.

Blood samples were collected at 0, 7^th^, and 14^th^ d. Blood was taken from the tail vein, and centrifuged immediately at 1,500×g for 15 min at 4°C to obtain serum. Serum samples were stored at −80°C until analyses. The levels of serum UA, UN, and Cr were detected according to the methods described previously [28].

### Statistical analysis

Statistical analysis was performed by using SAS 9.1 system (SAS Institute Inc, USA) and GraphPad Prism 5 (Graph Pad Software, La Jolla, CA, USA). Data are presented as Means±S, *P*<0.05 was considered as significant.

## Results

### Screening of inosine and guanosine degrading LAB strains

A total of fifty-five LAB strains were isolated from different Chinese sauerkraut samples, and they were all tested for inosine and guanosine assimilating abilities based on HPLC detection. Results are summarized in Table 1. The majority of the tested fifty-five LAB strains exhibited purine assimilation abilities, with the average assimilation rate of 11.30% for inosine, and 10.20% for guanosine. Among them, strains DM9218, DM9242, and DM9505 were found to assimilate purine nucleosides at greater than 1.00×10^−3^ g/L/min, and assimilation rates over 25.00%. In particular, DM9218 showed the highest rates of 99.31% and 99.64%, for inosine and guanosine respectively. These three stains were selected for further study.

**Table 1:** The abilities of candidate LAB strains to assimilate inosine and guanosine.

Strain	*V*_ino_ (g/L/min)	α_ino_ (%)	*V*gua (g/L/min)	α_gua_ (%)	Strain	*V*_ino_ (g/L/min)	α_ino_ (%)	*V*gua (g/L/min)	α_gua_ (%)
DM9010	1.14E-04	1.96	2.63E-04	4.53	DM9234	2.68E-04	4.60	1.10E-04	1.89
DM9013	3.95E-04	6.78	6.02E-04	10.38	DM9237	1.36E-04	2.33	3.67E-04	6.33
DM9014	6.77E-04	11.61	6.95E-04	11.98	DM9241	4.82E-04	8.27	6.92E-04	11.93
DM9017	7.88E-04	13.52	8.63E-04	14.88	DM9242	1.31E-03	22.47	1.58E-03	27.18
DM9019	4.43E-04	7.60	3.96E-04	6.83	DM9247	5.18E-04	8.89	1.00E-03	17.31
DM9023	4.82E-04	8.27	2.23E-04	3.84	DM9250	4.62E-04	7.92	9.82E-04	16.93
DM9037	2.44E-04	4.19	3.54E-04	6.10	DM9258	3.64E-04	6.24	4.95E-04	8.54
DM9040	9.04E-04	15.51	8.62E-04	14.86	DM9500	9.03E-04	15.49	9.03E-05	1.55
DM9059	2.41E-04	3.89	3.05E-04	5.26	DM9501	7.53E-04	12.92	9.63E-05	1.72
DM9065	1.87E-04	3.21	9.32E-05	1.61	DM9502	7.97E-04	13.66	2.79E-04	4.81
DM9077	5.97E-04	10.25	8.22E-05	1.42	DM9503	6.53E-04	11.20	1.21E-04	2.01
DM9091	7.76E-04	13.31	7.31E-04	12.59	DM9504	9.18E-04	15.75	7.71E-04	13.30
DM9125	3.36E-04	5.77	7.45E-04	12.85	DM9505	1.74E-03	29.85	1.76E-03	30.37
DM9155	4.87E-04	8.35	7.31E-04	12.60	DM9506	9.70E-04	16.64	3.15E-04	5.43
DM9157	3.31E-04	5.67	2.50E-04	4.31	DM9507	3.29E-04	5.63	1.63E-04	2.82
DM9166	2.71E-04	4.64	6.13E-04	10.58	DM9508	6.65E-04	11.41	2.36E-04	4.07
DM9168	3.88E-04	6.64	6.95E-04	11.99	DM9509	7.91E-04	13.58	3.37E-04	5.77
DM9172	3.22E-04	5.52	2.50E-04	4.31	DM9510	1.57E-04	2.69	5.67E-05	0.99
DM9176	2.69E-04	4.61	4.13E-04	7.12	DM9514	1.20E-03	20.58	8.02E-04	13.82
DM9183	3.95E-04	6.77	7.76E-04	13.38	DM9515	7.44E-04	12.75	9.87E-05	1.74
DM9185	4.53E-04	7.77	6.53E-04	11.26	DM9517	7.08E-04	12.14	2.59E-04	4.46
DM9194	3.67E-04	6.29	5.61E-04	9.67	DM9518	9.83E-04	16.86	6.02E-04	10.38
DM9200	6.53E-04	11.20	6.34E-04	10.93	DM9519	5.99E-04	10.27	8.12E-05	1.39
DM9206	4.47E-04	7.66	3.21E-04	5.53	DM9520	7.76E-04	13.31	9.20E-05	1.61
DM9207	2.45E-04	4.21	5.18E-04	8.93	DM9521	3.27E-04	5.61	6.17E-04	10.63
DM9213	5.48E-04	9.40	9.30E-04	16.03	DM9529	5.93E-04	10.18	1.52E-04	2.62
DM9218	5.79E-03	99.31	5.78E-03	99.64	DM9530	6.84E-04	6.39	6.67E-07	0.01
DM9233	6.97E-04	11.96	9.77E-04	16.85	DM9218-A	5.79E-03	99.48	5.81E-03	99.73

Values are Means, n = 3. *V*_ino_, the assimilating speed of inosine by LAB strain; α_ino_, the assimilating rate of inosine by LAB strain; *V*_gua_, the assimilating speed of inosine by LAB strain; α_gua_, the assimilating rate of inosine by LAB strain. Underlines indicate strains with assimilating speed higher than 1.00×10^−3^ g/L/min. DM9218-A, the acclimated DM9218 strain.

**Figure 1:**
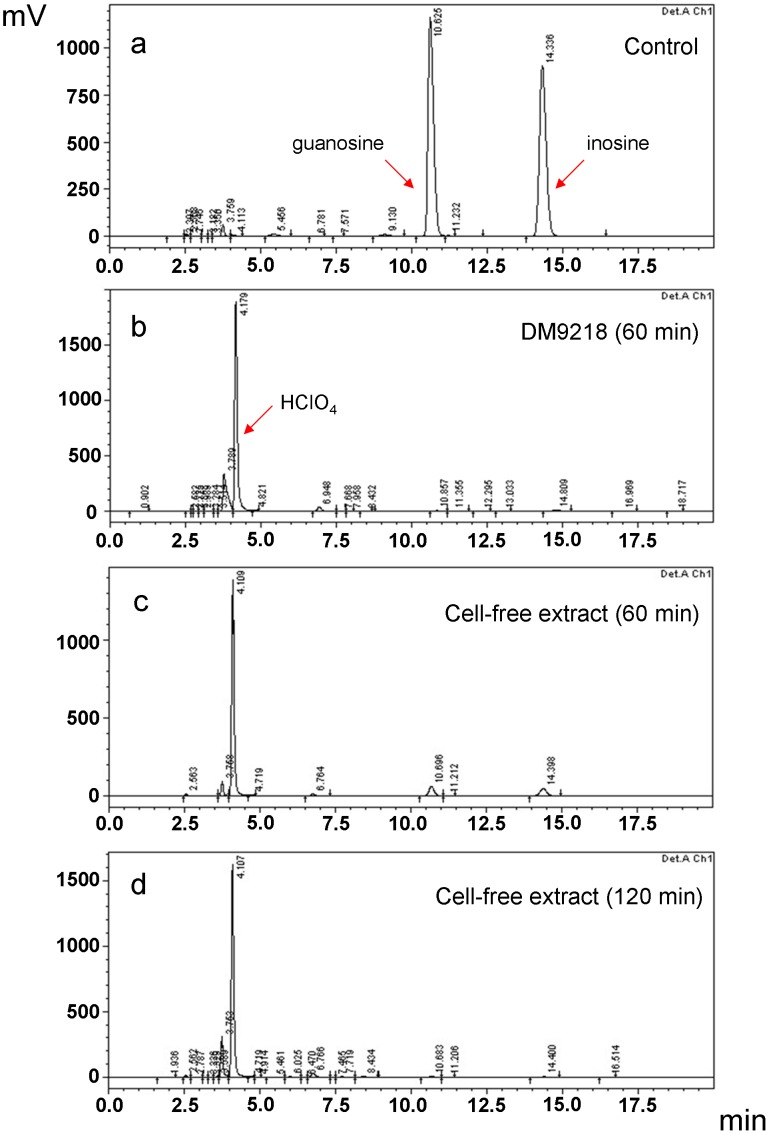
The degradation of inosine and guanosine by DM9218. (a) Control: inosine and guanosine solution without inoculation of bacteria (270 µL of the inosine and guanosine solution was incubated at 37°C for 60 min, after that 30 µL HClO_4_ was added, 20 µL of the mixture was analyzed by HPLC). (b) DM9218 living cells were incubated with inosine and guanosine solution for 60 min. (c) Cell-free extracts of DM9218 were incubated with inosine and guanosine solution for 60 min. (d) Cell-free extracts of DM9218 were incubated with inosine and guanosine solution for 120 min. See details in methods.

To clarify whether the purine compounds were degraded by LAB, cell-free extracts of DM9218 were incubated with the inosine and guanosine solution for 60 and 120 min, and the concentration of purine compounds analyzed by HPLC. Results in Fig. 1 show that after 60 min incubation, the concentration of inosine and guanosine was decreased by 85.76% and 86.20%, respectively (Fig. 1c). After 120 min incubation, the two purine compounds were degraded by 98.81% and 98.98% respectively (Fig. 1d), which was comparable with purine degradation by living DM9218 cells (Fig. 1b). Cell-free extracts of DM9242 and DM9505 were also incubated with inosine and guanosine for 120 min. 20.28% and 25.16% degradation of inosine and 24.25% and 28.97% degradation of guanosine were observed for DM9242 and DM9505 respectively.

### Evaluation of the probiotic potential of DM9218, DM9242 and DM9505

The tolerance of the three candidate strains to acid and bile salt are summarized in Table 2, which shows there was little variation among the candidate LAB strains. The acid tolerance test revealed that strains DM9505 and DM9218 could survive at pH values as low as 2, but DM9242 could only survive at a pH over 3. The 3 strains were not tolerant to bile salt. When the medium contained 0.2% bile salt, DM9218 and DM9242 could grow weakly, but DM9505 could not grow at all. When the concentration of bile salt was increased to 0.3%, no strains grew. All the three strains could grow well in media supplemented by 0.59∼1.48 µg/mL pepsin or 33.60∼72.32 U/g trypsin.

**Table 2:** The tolerance of candidate LAB strains to biological barriers (Log CFU/mL).

Strains	Initial counts^a^	pH			Bile salt (%)		
		2	3	4	0.1	0.2	0.3
DM9218	8.59±0.18	4.36±0.10	6.25±0.13	9.05±0.16	2.86±0.13	-	-
DM9242	9.02±0.11	-b	2.56±0.24	3.12±0.14	1.36±0.18	-	-
DM9505	8.98±0.23	3.11±0.23	5.87±0.18	8.15±0.24	-	-	-

Strains	Initial counts^a^	Pepsin (µg/mL)			Trypsin (U/g)		
		0.59	0.72	1.48	33.60	52.96	72.32
DM9218	8.59±0.18	9.24±0.47	9.44±0.52	8.99±0.46	8.98±0.61	9.13±0.41	9.04±0.48
DM9242	9.02±0.11	9.63±0.33	9.21±0.18	9.02±0.84	9.07±0.19	9.16±0.11	8.99±0.71
DM9505	8.98±0.23	9.01±0.13	9.11±0.87	9.11±0.77	9.04±0.27	9.00±0.53	8.99±0.37

a Each value represents Mean±S, n = 3.

b No growth.

**Figure 2:**
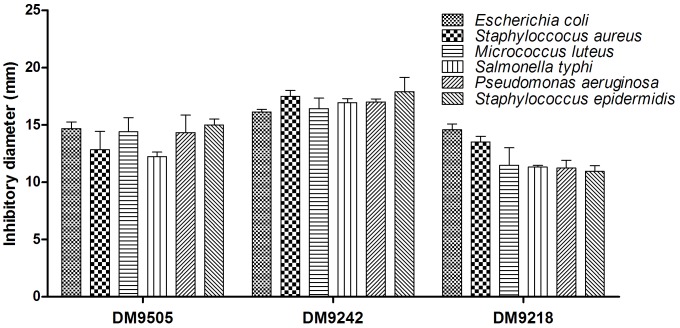
The antimicrobial activities of DM9218, DM9242, and DM9505. All values are Means ± S, n = 3.

The antimicrobial activities of strain DM9218, DM9242 and DM9505 are shown in Fig. 2. All strains exhibited specific inhibitory activities against some of the pathogenic microorganisms tested. Strain DM9242 showed strong inhibitory activities especially to *Salmonella typhi* and *Staphylococcus aureus*. Strain DM9505 strongly inhibited *Pseudomonas aeruginosa*. Strain DM9218 showed high inhibitory activities to *Escherichia coli* and *Staphylococcus aureus*.

**Figure 3:**
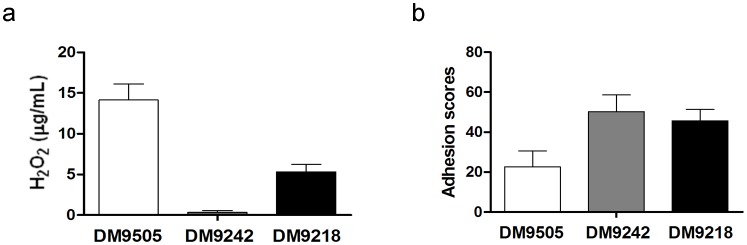
Production of H_2_O_2_ by DM9218, DM9242 and DM9505, and their adhesion abilities to Caco-2 cells. (a) Production of H_2_O_2_ by strains in cold, sterile phosphate buffer (pH 6.5) incubated at 5°C for 5 days. (b) Adhesion abilities to Caco-2 cells. The adhesion scores indicate values of LAB cells adhered to one Caco-2 cell. All values are Means ± S, n = 3.

The H_2_O_2_ content produced by candidate strains in cold, sterile phosphate buffer (pH 6.5) was determined (Fig. 3a). DM9505 and DM9218 were found to produce 14.17 µg/mL and 5.32 µg/mL of H_2_O_2_, respectively, while minimal H_2_O_2_ was detected in the phosphate buffer inoculated with DM9242.

The cell adhesion ability of the candidate strains to human enterocyte-like Caco-2 cells was tested. Results are shown in Fig. 3b. DM9242 showed the highest adhesion value of 50.1±8.35 cells/Caco-2 cell. This was followed by DM9218, with an adhesion value of 45.5±5.67, and DM9505 with an adhesion value of 22.7±7.86.

The sensitivity of the three strains to antibiotics (Table 3) was tested by disc diffusion assay against 16 different antibiotics from 9 categories. The results demonstrated that the strains were susceptible to antibiotics belonging to the penicillins (including penicillin, piperacillin, and amoxicillin), cephalosporins (cephalothin), macrolides (erythromycin), and ansamycins (rifampicin). They were found to be resistant to the aminoglycosides (gentamicin), quinolones (levofloxacin, nalidixic acid, ciprofloxacin), and glycopeptides (vancomycin). The three strains showed intermediate sensitivity to the other antibiotics tested.

### Identification of DM9218 by 16S rDNA sequencing

**Figure 4:**
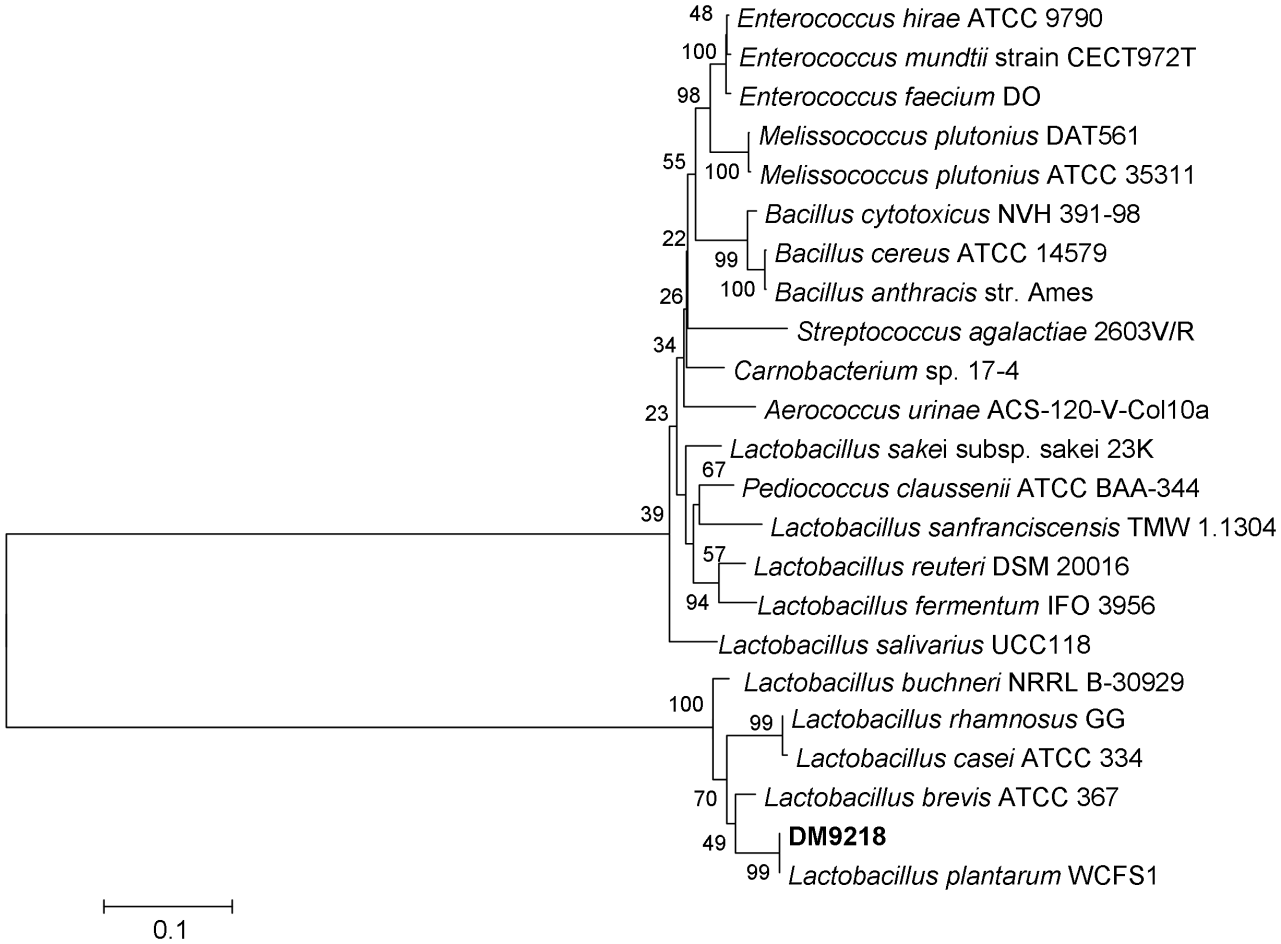
Phylogenetic Neighbor-Joining tree of DM9218 based on 16S rDNA sequences. The phylogenetic tree was constructed using the neighbor-joining method by MEGA 6.0 based on 1372 bp of the 16S rDNA sequences. The numbers at the nodes are bootstrap confidence levels (percentage) from 1,000 replicates. The scale bar represents 0.1 substitutions per nucleotide position. Reference sequences were obtained from the GenBank nucleotide sequence database (NCBI).

Owing to its superior performance in degrading inosine and guanosine, and its superior probiotic characteristics compared with the two other candidates, DM9218 was selected for further study. It was first identified by 16S rDNA sequencing. The 16S rDNA sequence of DM9218 had the highest identity (99%) with the 16S rDNA of *Lactobacillus plantarum* WCFS1 [29] (Fig. 4). It also showed high similarity with the 16S rDNA of *Lactobacillus brevis* ATCC 367 (95%) [30] and *Lactobacillus rhamnosus* GG (93%) [31].

### Acclimation of DM9218 to bile salt

**Figure 5:**
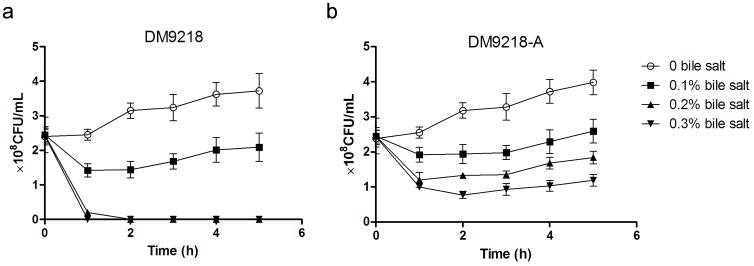
The growth of DM9218 and DM9218-A in bile salt containing MRS media. (a) The growth of DM9218 in bile salt-containing media. (b) The growth of DM9218-A (acclimated DM9218) in bile salt containing media. 10^8^ CFU/mL of freshly prepared LAB were inoculated into 10 mL of the autoclaved solutions and incubated at 37°C for 1∼5 h before viable plate counting. All values are Means ± S, n = 3.

To overcome the poor tolerance to bile salt, we acclimatized DM9218 to bile salt by cultivating it on MRS media containing increasing concentrations (0.1–0.3%) of bile salt. After ten generations, the clones growing on 0.3% bile salt containing MRS agar media were picked, and their growth pattern in liquid media with different concentrations of bile salt tested. Compared with DM9218, the acclimated strain (DM9218-A) showed no difference when grown in MRS medium without adding bile salt (Fig. 5a and b). However, it showed tolerance to 0.2% and 0.3% bile salt, the percentage survival increasing to 75.10% and 48.97% respectively after 5 hours incubation. Furthermore, the inosine and guanosine degradation abilities of DM9218-A were not affected (See Table 1).

### Detection of DM9218-A in rats

**Figure 6:**
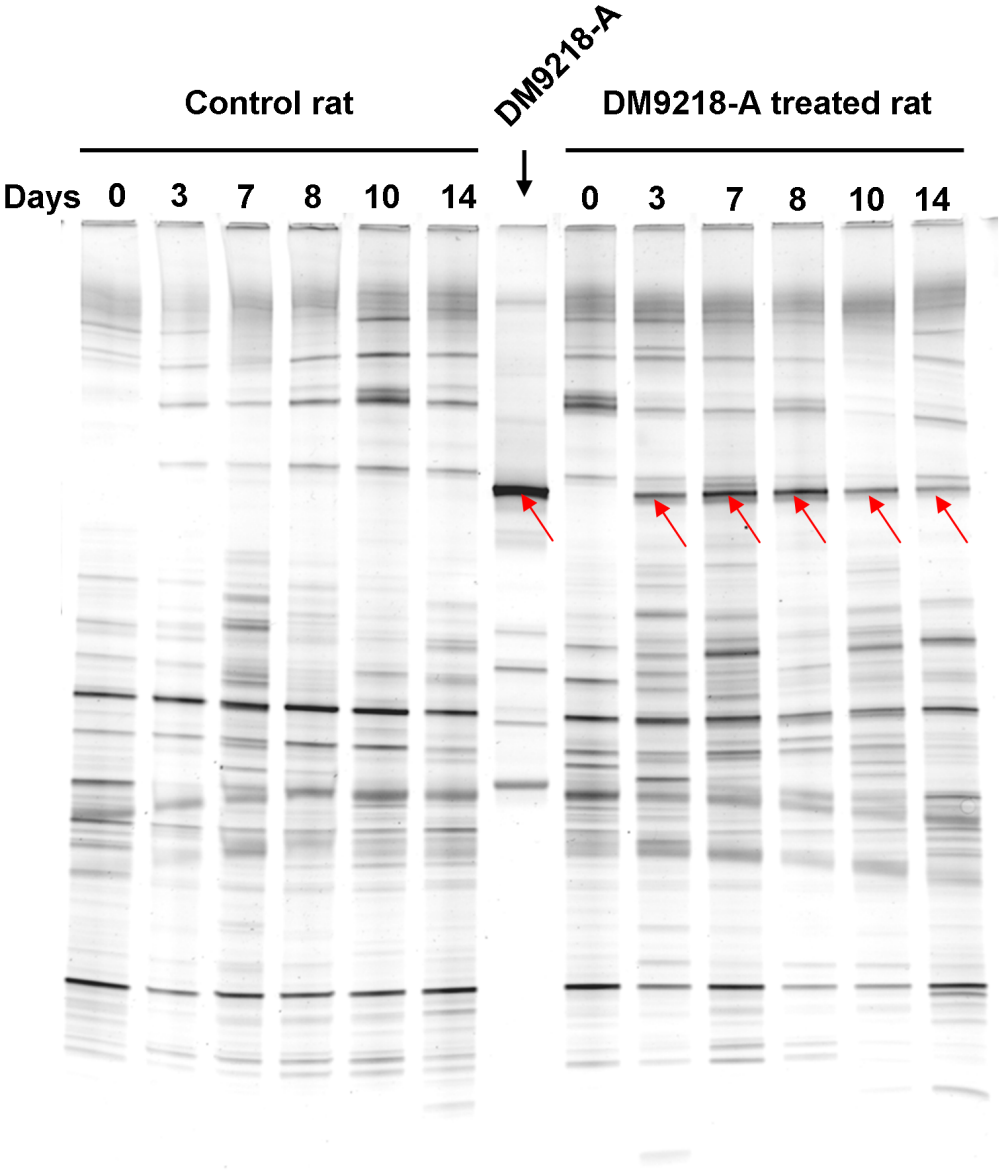
Detection of DM9218-A in rats. The control rats (n = 7) was given food and water *ad libitum*. The DM9218-A treated rats (n = 7) were first intragastrically administrated DM9218-A cell solution (1.2×10^9^ CFU/mL in 0.85% NaCl, 1 mL per day) for 7 days, and then given food and water *ad libitum* for 7 days. DGGE profiles of V3 region of 16S rRNA gene amplicons obtained in PCRs with F338/R518 set of primers. Red arrows indicate eluted and sequenced bands. Lanes indicate the DM9218-A pure culture, and the faecal contents of DM9218-A treated or untreated rats taken at indicated sampling days.

To investigate whether DM9218-A can survive in the gastrointestinal (GI) tract of rats, feces taken from rats before and after intragastric administration were analyzed as an indicator of gut microbiota composition. The DGGE profile of 16S rRNA gene amplicons obtained from a pure culture of DM9218-A strain revealed the existence of several copies of the 16S rRNA gene, with one copy staining most intensively on the gel. DGGE analysis of 16S rRNA products obtained from fecal contents of DM9218-A treated rats (3 to 14 days samples after administration) revealed the band corresponding to the dominant band of 16S rDNA from DM9218-A (Fig. 6). Suspected bands, as well as the dominant pure culture band, were sequenced queried against the NCBI genome collection. The results confirmed that all eluted bands belonged to *Lactobacillus plantarum*. No band corresponding to the dominant pure DM9218-A culture band was detected in control rats or fecal content of rats before treatment. Along with the demonstration of the ability of DM9218-A to survive in the GI tract of the rats, the results obtained by DGGE analysis showed no significant shift in the intestinal microbial community of the treated rats.

### Effects of DM9218-A on hyperuricemia in rats

**Figure 7:**
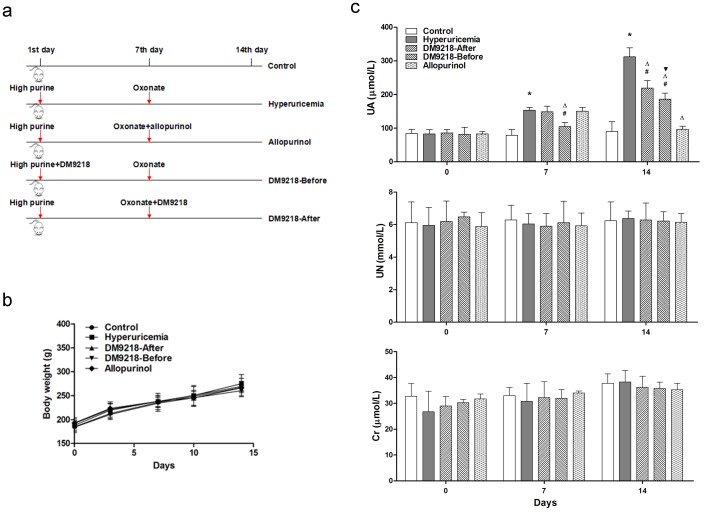
The animal test. (a) The outline of animal test. (b) The body weights of rats were measured randomly at day 0, 3, 7, 10, and 14. Data are Means ± S, n = 8. No differences in body weight gain were detected among the different experimental groups. (c) The level of serum UA (uric acid), * means *P*<0.05 compare with control rats, ▵ means *P*<0.05 compare with hyperuricemic rats, # means *P*<0.05 compare with allopurinol treated rats, ▾ means *P*<0.05 compare with DM9218-After group. (d) The level of serum UN (urea nitrogen). (e) The level of serum Cr (creatinine). All values are Means ± S, n = 8.

To examine the effect of DM9218-A on the serum uric acid (UA) level of hyperuricemic rats, we induced hyperuricemia by intraperitoneal injection of potassium oxonate into rats, supplemented with a high purine diet. Fig. 7a shows the outline of the animal study: one group of rats was intragastrically administrated DM9218-A from the very beginning of hyperuricemia induction, they were considered the preventive DM9218-Before group. After 7-days, 4 groups (all except the control group) were injected with potassium oxonate for 7 days, 2 groups were treated with DM9218-A or allopurinol, and they were defined as the DM9218-After and allopurinol groups respectively. No differences in food consumption (data not shown) and body weight (Fig. 7b) were detected among the experimental groups during the trial (*P*>0.05). Blood samples were taken on days 0, 7, and 14. Fig. 7c (top) shows that after 7 days' high purine diet, the blood UA level of hyperuricemic rats increased to 153.25 µmol/L, which is significantly higher than control rats (79.00 µmol/L, *P* = 0.0016), 14 days after high purine diet combined with 7 days potassium oxonate injection, the blood UA level of hyperuricemic rats reached to 311.75 µmol/L, which is over 3.45 fold that of control rats (*P* = 0.0001). It can therefore be considered that under these experimental conditions, hyperuricemia was successfully established. The administration of DM9218-A to rats from the first day (DM9218-Before group) resulted in a dramatically lower level of blood UA compared with the control group at the 14^th^ day (*P* = 0.0015); however this level was still higher than that of healthy controls (*P* = 0.0113). From the 8^th^ day, we treated the hyperuricemic rats with allopurinol. Results of the 14^th^ day samples indicated that allopurinol significantly reduced blood UA to a healthy level (*P*>0.05 compared with healthy control). The blood UA level in rats administrated DM9218-A from the 8^th^ day were lower than the hyperuricemic group (*P* = 0.0211), but still higher than the healthy group (*P* = 0.0454). The preventive treatment of DM9218-A caused a greater reduction in serum UA concentration as compared with the DM9218-After group (*P* = 0.0487). Effects of DM9218-A on blood urea nitrogen (UN) and creatinine (Cr) contents of rats were also evaluated. Fig. 7c (middle and bottom) shows that no significant differences were detected among the control group, hyperuricemia model group and the rats treated with DM9218-A or allopurinol (*P*>0.05).

## Discussion

Purine rich foods such as meat and seafood, as well as alcoholic beverages potently exacerbate hyperuricemia, the major factor causing gout. Over the past ten years, more and more studies have documented the biological interference of uric acid with other diseases, such as hypertension, to initiate endothelial dysfunction, vascular damage and renal disease [32], [33]. Normalizing serum uric acid level is thus absolutely essential to reduce the risk of complications. However, prescribing uric acid-lowering drugs to individuals with asymptomatic hyperuricemia is not recommended by current policy because of insufficient evidence [32]. Therefore, probiotics with the ability to degrade purine compounds in food deserve to be investigated exhaustively for the prevention of hyperuricemia.

LAB have been investigated intensively over the past few decades as they were found to play important roles in gastrointestinal transit and food processing [34]. Many of them were proven to have characteristics beneficial to human health [20]. Chinese sauerkraut, the typical representative of Chinese traditional fermented foods, is a rich natural source of LAB. Many species of LAB have been isolated from Chinese sauerkraut and used as probiotics [35]. The objective of this study was to isolate a collection of probiotic LAB from Chinese sauerkraut, screen for purine compound degrading strains and evaluate the potential of the candidate strains for the prevention and treatment of hyperuricemia.

Most of the fifty-five isolates were found to assimilate purine nucleosides at different speeds, with three of them possessing high assimilation capabilities. However it was still unclear whether the purine nucleosides were degraded or incorporated into cells. To clarify this, the degradation of inosine and guanosine by cell-free extracts of DM9218, DM9242 and DM9505 were analyzed. The results showed that the cell-free extracts were able to degrade the purine compounds, and the degradation rates (during 120 min incubation) were comparable to that of living LAB cells.

Microorganisms degrade nucleosides mainly through the biosynthesis of nucleoside hydrolases. These nucleosidases are widely found in plants and microorganisms but have not yet been detected in mammals [36]. Nucleoside hydrolases break the β-glycoside bonds of nucleosides and release nitrogenous bases and pentose. The correlation between inosine and guanosine assimilating abilities of the candidate strains were tested by SAS system and showed that there is a strong positive correlation between the assimilation of inosine and guanosine (Fig. S1). This suggests the existence of nucleoside hydrolases in the tested LAB strains.

To determine whether the tested strains can survive in the gastrointestinal environment and colonize successfully, the biological characteristics of the three candidate strains were evaluated, including tolerance to acid and bile salt, antimicrobial activity, sensitivity to drugs and cell adhesion ability. Results showed that all strains possess certain inhibitory activities against the tested pathogenic bacteria. In general, the antibacterial ability of LAB is derived from the main characteristics: the production of lactic acid by fermentation of sugars, lowering the pH of the environment, making it unsuitable for the growth of other bacteria; and the production of antibacterial substances, such as H_2_O_2_ and bacteriocins. Testing of the H_2_O_2_-producing ability of the candidate strains suggested that there is no clear relationship between their antibacterial activities and the production of H_2_O_2_, it is likely that the inhibition is due to the accumulation of organic acids. The three candidate strains all have moderate tolerance to acid, but poor tolerance to bile salt. DM9218 and DM9242 can survive in 0.1% bile salt containing medium, but DM9505 could not even grow in 0.1% bile salt containing medium. Adhesion to the intestinal epithelium is an important requisite for allowing probiotics to modulate the immune system. Therefore the cell adhesive abilities of the three strains to Caco-2 cells were also evaluated. According to Maccaferri and colleagues [37], more than 40 bacterial cells adhered to one Caco-2 cell is defined as strongly adhesive; thus, DM9242 and DM9218 can be classified as strongly adhesive strains, while the adhesive ability of DM9505 is relatively weak. Considering the superior purine nucleosides degrading ability and moderate biological characteristics of strain DM9218, it was chosen as the optimal strain for further study.

Bile is secreted by the liver cells and aids the digestion of lipids in the small intestine. It has a strong inhibitory effect on intestinal microbiota, especially on Gram positive bacteria. Therefore, the ability to survive in a bile salt containing environment is a prerequisite condition for DM9218 to exert its biological activity *in vivo*. Because of its poor tolerance to bile salt, we decided to acclimatize DM9218 to bile salt. According to the industrial standard for the attenuation of LAB strains, we gradually acclimatized DM9218 from 0 to 0.3% bile salt containing media. After acclimation, the DM9218-A strain showed increased tolerance to 0.2% and 0.3% bile salt, while its purine degrading ability was not affected.

The survival of DM9218-A in the GI tract of rats was determined by PCR-DGGE. The positive PCR-DGGE result provided evidence that DM9218-A successfully survived in the digestive tract of rats during the process of intragastric administration, as the DNA of dead bacteria is degraded by nucleases released in the GI tract [38]. After the administration of DM9218-A stopped, the intestinal population of DM9218-A decreased (14^th^ day), although it was still detectable. Moreover, DM9218-A treated rats displayed a microbial profile that was similar to untreated rats, which suggests that no perturbation of the host gut microbiota will be caused by intragastric administration of DM9218-A.

It is currently unclear whether the intake of purine compounds can cause an elevation of serum uric acid. However, it has been difficult to establish an animal hyperuricemia model to test this [39], because commonly used laboratory animals such as rats, mice and rabbits, all express urate oxidase. This enzyme oxidizes the poorly soluble uric acid to water soluble allantoin, thus it is very difficult to induce hyperuricemia in these animals. As for humans and apes, mutation of the liver uricase gene resulted in inactivation of uric acid oxidase [40], therefore the occurrence of hyperuricemia in human is much higher than other mammalian. The methods of inducing hyperuricemia in rats include gene knockout, high purine diet, injection of potassium oxonate and the combination of high purine diet with potassium oxonate injection. The combination method can efficiently elevate serum uric acid in rats, and compared with potassium oxonate or high purine diet alone, the effect is greater and more stable and the duration of hyperuricemia longer. We therefore adopted this method to induce hyperuricemia in rats.

After injection of potassium oxonate and high purine diet, the level of serum uric acid in treated rats was elevated more than 2.45 fold compared with controls. Application of DM9218-A to the hyperuricemic rats significantly decreased the uric acid level, but it was still higher than the allopurinol treated group. This is because allopurinol directly inhibits xanthine oxidase, a key enzyme in the purine metabolite pathway, which blocks the production of uric acid. In contrast, DM9218-A competes with the intestinal epithelium for the absorption of nucleosides in food. Therefore there are still free purine bases that can be absorbed by the epithelium and eventually metabolized into uric acid. The experimental results also suggest that, once applied to humans, the preventive effect of DM9218-A against hyperuricemia may be stronger than the effect of treatment after hyperuricemia has been induced. No significant differences in the level of serum creatinine and urea nitrogen were observed during the two week study period, which suggests that, because of the short modeling time, no serious renal injury has been induced in the rats.

## Conclusions

Although further systemic safety assessment is necessary, our results suggest that DM9218-A is a promising candidate as an adjunctive treatment in patients with hyperuricemia, especially during the onset period of disease. DM9218-A also has potential as a probiotic in the prevention of hyperuricemia in the normal population.

## Supporting Information

Figure S1 **The correlation between abilities of tested strains to assimilate inosine and guanosine.** The assimilation abilities of candidate strains were tested by SAS 9.1 System for the Pearson correlation coefficients.
(TIF)
