# Supplementary figures and images for: Screening and Characterization of Purine Nucleoside Degrading Lactic Acid Bacteria Isolated from Chinese Sauerkraut and Evaluation of the Serum Uric Acid Lowering Effect in Hyperuricemic Rats
Source: PLoS One. 2014 Sep 3;9(9):e105577. doi: 10.1371/journal.pone.0105577 (PMC4153548; doi:10.1371/journal.pone.0105577)

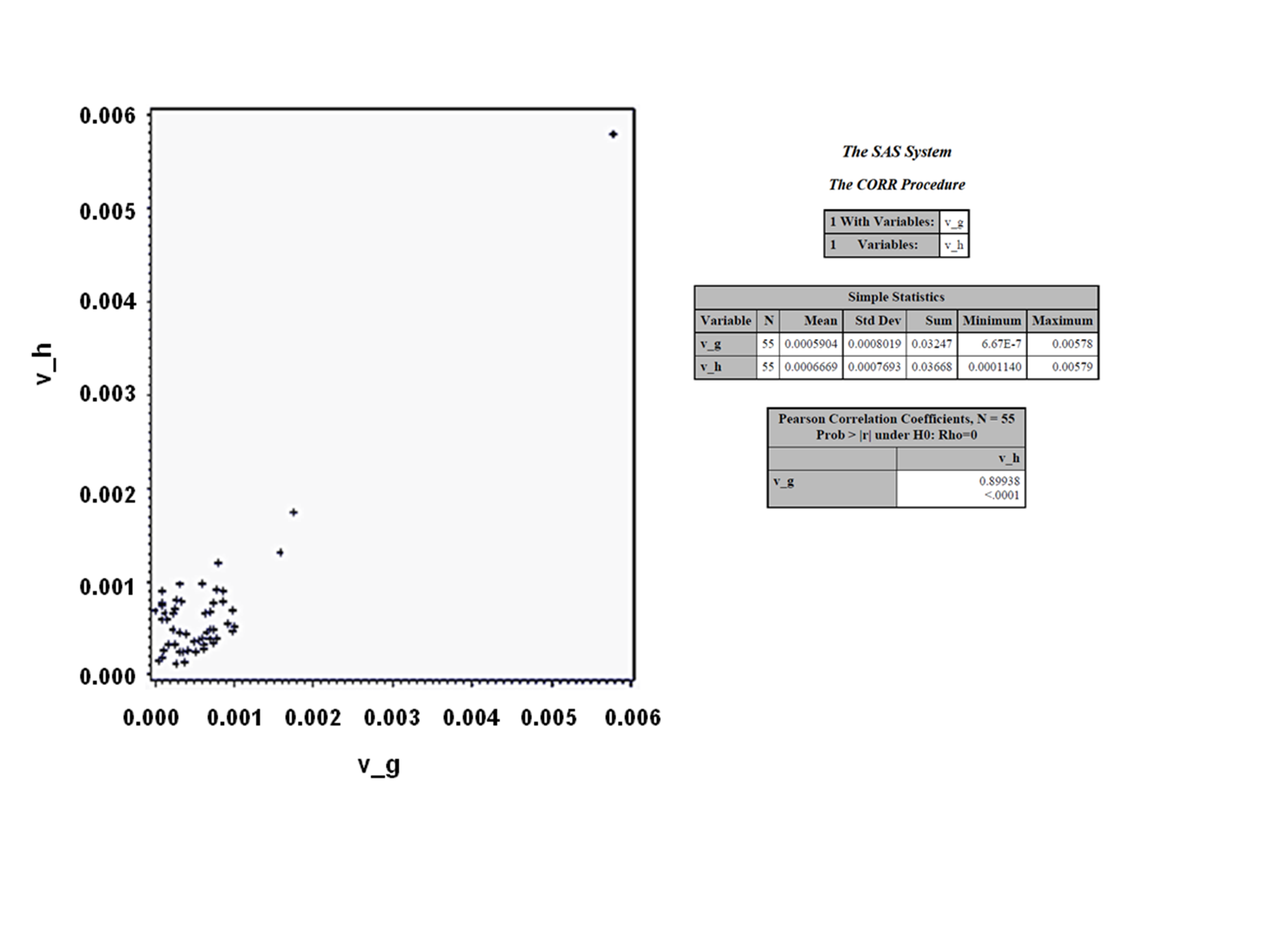

Supplement: Figure S1 — The correlation between abilities of tested strains to assimilate inosine and guanosine. The assimilation abilities of candidate strains were tested by SAS 9.1 System for the Pearson correlation coefficients. (TIF) [file pone.0105577.s001.tif]
